# Development and validation of a novel fibroblast scoring model for lung adenocarcinoma

**DOI:** 10.3389/fonc.2022.905212

**Published:** 2022-08-22

**Authors:** Shiyou Wei, Xuyu Gu, Wentian Zhang

**Affiliations:** ^1^ Department of Anesthesiology, Shanghai Pulmonary Hospital, School of Medicine, Tongji University, Shanghai, China; ^2^ School of Medicine, Southeast University, Nanjing, China; ^3^ Department of Thoracic Surgery, Shanghai Pulmonary Hospital, School of Medicine, Tongji University, Shanghai, China

**Keywords:** lung adenocarcinoma, cancer-associated fibroblasts, tumor microenvironment, scoring model, prognosis

## Abstract

The interaction between cancer-associated fibroblasts (CAFs) and the tumor microenvironment (TME) is a key factor for promoting tumor progression. In lung cancer, the crosstalk between CAFs and malignant and immune cells is expected to provide new directions for the development of immunotherapy. In this study, we have systematically analyzed a single-cell dataset and identified interacting genes between CAFs and other cells. Subsequently, a robust fibroblast-related score (FRS) was developed. Kaplan-Meier (KM) and ROC analyses showed its good predictive power for patient prognoses in the training set comprising of specimens from the cancer genome atlas (TCGA) and in three external validation sets from the Gene Expression Omnibus (GEO). Univariate and multivariate Cox regression analyses suggested that FRS was a significant prognostic factor independent of multiple clinical characteristics. Functional enrichment and ssGSEA analyses indicated that patients with a high FRS developed “cold” tumors with active tumor proliferation and immunosuppression capacities. In contrast, those with a low FRS developed “hot” tumors with active immune function and cell killing abilities. Genomic variation analysis showed that the patients with a high FRS possessed a higher somatic mutation burden and copy number alterations and were more sensitive to chemotherapy; patients with a low FRS were more sensitive to immunotherapy, particularly anti-PD1 therapy. Overall, these findings advance the understanding of CAFs in tumor progression and we generated a reliable FRS-based model to assess patient prognoses and guide clinical decision-making.

## Introduction

Lung cancer has the highest incidence among all cancer types and is the leading cause of cancer-related deaths (1); lung adenocarcinoma (LUAD) is its most common histological type. Several epidemiological investigations and experimental studies have attributed the onset and progression of LUAD primarily to environmental factors and genetic alterations (2–4). Given a large number of non-smokers with LUAD, previous theories based solely on environmental factors have been disproven and research attention has been re-focused on profound alterations in the genetic content. To date, there are two main genetic factor-related treatment strategies, namely, targeted therapy and immunotherapy (5). However, most patients who receive targeted therapy are prone to resistance, and only a minority of them may benefit from immunotherapy. Therefore, it is crucial to develop robust tools for prognostic prediction and assessment of treatment responses to further facilitate accurate diagnoses and devise individualized treatment strategies.

Tumor microenvironment (TME) is defined as the environment surrounding the tumor, including the extracellular matrix, immune cells, and stromal cells, all of which are closely associated with tumor progression and treatment outcomes (6). Accumulating evidence elucidate the role of TME infiltration in immune therapeutic responses and resistance against different cancer types; these studies have also investigated their impact on patient prognoses (7, 8). Previous studies have focused more on immune cells. However, several findings have now highlighted the importance of stromal cells in tumor progression (9, 10). Cancer-associated fibroblasts (CAFs), a representative component of stromal cells, play crucial roles in cancer genesis, progression, and invasion (11, 12). Recently, the interaction between CAFs and the tumor immune microenvironment (TIME) has been identified as a key factor in promoting tumor progression (13, 14). CAFs interact with immune cells and other immune components within the TIME through various secreted cytokines, growth factors, and chemokines, resulting in an immunosuppressive TME that allows cancer cells to evade the surveillance mechanisms of the immune system (14, 15). Therefore, further investigation into the crosstalk between CAFs and TME is expected to provide new strategies for LUAD treatment, in particular for immunotherapy.

In this study, we used the single-cell dataset, GSE131907, to evaluate the crosstalk between CAFs and other cells. In addition, receptor-ligand pairs were systematically identified for interactions of CAFs with other cells. Based on these receptor-ligand genes, we generated the fibroblast-associated score (FRS) using the LASSO algorithm in the TCGA-LUAD cohort and the GEO meta-cohort to predict patient prognoses and estimate their sensitivity to chemotherapy and immunotherapy. Additionally, the associations among FRS, biological functions, TIME, and genomic alterations were systematically assessed. In summary, our findings are expected to advance the understanding of CAF functions in cancer as we have constructed and described here a robust scoring system to accurately predict patient prognoses and guide clinical decision-making.

## Material and methods

### Data extraction from online databases

The single-cell transcriptome dataset, GSE131907, was extracted from the GEO database (https://www.ncbi.nlm.nih.gov/geo/), consisting of data of 58 sequences from 44 patients. Next, these data were processed using the 10x Genomics method. Of these, we selected 29 normal lung tissues and early, advanced, and brain-metastasized lung tissues for further analyses. Detailed data processing procedures and ethical approval have been described previously (16).

The data of transcriptome RNA sequencing, Mutect2 mutation, HumanMethylation450 array, copy number variations (CNVs), and the corresponding clinical information were downloaded from TCGA database (https://cancergenome.nih.gov/) using the GDC API. A total of 492 LUAD samples were collected after the exclusion of patients with missed visits and incomplete clinical information. The raw FPKM sequencing data were normalized by TPM and used as the training cohort. Three mature LUAD cohorts were collected from GEO, including dataset GSE30219 from the Affymetrix HG-U133 Plus 2.0 Array platform, dataset GSE72094 from the Rosetta/Merck Human RSTA Custom Affymetrix 2.0 platform, and dataset GSE42127 from the Illumina HumanWG-6 v3.0 expression bead chip. To prevent batch effects on these chips, we merged the three GEO datasets and normalized the data by the log2 transformation using the combat function of the “sva” package (17). Subsequently, LUAD meta-data containing the complete clinical information of 615 individuals were used as the validation cohort. Additionally, we collected the publicly available immunotherapy cohorts with complete clinical information and transcriptomic data. Finally, the information of a cohort of advanced uroepithelial carcinoma treated with anti-PD-L1 immunotherapy (Imvigor210) consisting of 298 patients (8) and a cohort of non-small cell lung cancer (NSCLC) of 27 patients treated with PD1 (GSE135222) was collected.

### Single-cell data analysis

The R package, “Seurat”, was used to process the scRNA-seq data. In addition, cells with “min.cells < 3” and “min.features < 200” were excluded. After filtering out the cells with > 60% mitochondrial sequencing count and nFeature_RNA > 7000, a total of 47822 cells were retained for subsequent analyses. The dataset was then normalized using the NormalizeData and ScaleData functions in Seurat. Cell types were identified according to the cell annotations provided in the original article.

To unravel the changes in the cell clusters during tumor progression, we used the R package, ‘monocle’, for the analysis of the single-cell trajectory. Subsequently, single-cell developmental trajectories were identified using the top 1500 variable genes (18). The Python package, “CellphoneDB”, was used to identify receptor-ligand exchanges between cell clusters; the receptor-ligand interactions between eight-cell clusters were thus identified at the molecular level (19). Receptor-ligand pairs with p-values < 0.05 were screened to assess the molecular interaction network among CAFs and other cells. Corresponding interacting genes were identified as fibroblast-related genes (FRGs). Finally, the GGplot2 package was used to visualize these results.

### Construction and validation of the FRS model

LUAD-TCGA cohort was used to train the model. Specifically, independent prognostic factors among FRGs were first screened by univariate Cox regression, and genes with P < 0.05 were included for further analysis. Subsequently, a Cox proportional risk model with LASSO penalties was used to identify the best prognostic model. To prevent overfitting, a five-fold cross-validation process was set up. Considering random sampling for cross-validation, 300 iterations were performed to identify the most stable prognostic model. The model with the highest frequency of occurrence in the 300 iterations served as the final prognostic model. Finally, FRS was calculated according to the following equation:


$$FRS=\sum i\;Coefficient\left(mRNA_{i}\right)\times Expression\left(mRNA_{i}\right)$$

To assess the predictive power of the risk scores in the training and validation sets, the consistency index (C-index) was calculated using the “survcomp” R package, with a larger C-index indicating a more accurate predictive power of model (20). Patients were classified into high- and low-risk groups based on the median FRS. Furthermore, the prognostic value of the risk model was systematically assessed using the Kaplan Meir (KM) survival curves, univariate and multivariate Cox regression analyses, and time-dependent ROC curves.

### Functional enrichment and immune infiltration analyses

We performed a single-sample gene set enrichment analysis (ssGSEA) based on the previously published molecular markers using the R package, “gsva”, to assess the activities of the biological pathways for the samples, including angiogenesis, epithelial-mesenchymal transition (EMT), myeloid inflammation, and molecular markers for other immune-related pathways (21–24). Molecular markers for hypoxia were collected from Msigdb (25). Detailed pathway-related gene markers are shown in **Table S1**. Additionally, GSEA was performed between high- and low-FRS groups, and the significant KEGG pathways were screened using the set threshold of P < 0.05. Moreover, functional enrichment of genes was obtained using the Metascape (www.metascape.org/) database.

The abundances of immune cell infiltrate in tumor samples were estimated using the R package, “CIBERSORT”, to evaluate the degree of infiltration of 22 immune cell types (26). The immune activity and tumor purity of the samples were assessed using the Estimate algorithm (27). The immunophenoscores (IPS) of the samples were calculated based on a previous study, with a higher IPS indicating a stronger immune activity of the sample (28). In short, IPS is calculated on a scale of 0-10 based on the transcriptome of the representative genes of the immunophenotype. Samplewise Z scores were positively weighted according to effective immune cells, negatively weighted according to inhibitive immune cells, and then averaged. Z score ≥ 3 is defined as IPS10, and Z score ≤ 0 is defined as IPS0.

Finally, homologous recombination deficiency (HRD) scores, indel neoantigens, and SNV neoantigens of the samples were obtained from Thorsson et al. (29).

### Comparison of genomic variation landscapes between two groups

To compare the differences in mutation burdens between the two groups, the mutation data were processed using the ‘maftools’ package in R. The total number of mutations in the samples was first calculated, and genes with a minimum number of mutations > 30 were identified. The differences in mutation frequencies between the high- and low-FRS groups were then compared using a chi-square test and visualized using maftools (30). CNV data were processed using the GISTIC 2.0 webtool in Genepattern. Subsequently, significantly amplified and missing chromosomal segments were identified and differences in CNVs on the chromosomal arms were assessed. Finally, these CNV results were visualized using the R package, “ggplot2”.

### Clinical significance of the risk model

The five most commonly used first-line drugs, including cisplatin, docetaxel, gemcitabine, paclitaxel, and vinorelbine, were selected for the treatment of LUAD. Ridge regression was used to calculate the half-maximal inhibitory concentration (IC50) for each sample, which was then used to assess the sensitivities of patients to chemotherapy in the high- and low-risk groups. Moreover, the accuracy of these predictions was assessed by a 10-fold cross-validation process (31). Furthermore, differentially expressed genes between the two groups were considered as potential therapeutic targets. The CMap database (https://clue.io/) was used to obtain the potential compounds targeting these genes. This database can not only predict drugs based on the gene expression profiles but also elucidate the mode of action (MoA) of these compounds targeting the corresponding molecular pathways. To assess the patient responses to immunotherapy, the TIDE online tool (http://tide.dfci.harvard.edu) was used (32). In addition, the unsupervised subclass mapping algorithm (https://cloud.genepattern.org/gp/) was used to assess the patient responses to anti-PD1 and anti-CTLA-4 immunotherapeutic regimens. Finally, we validated the predictive efficacy of FRS in the immunotherapy cohorts, Imvigor210 and GSE135222.

### Clinical specimens

We obtained 50 tissue specimens from patients who received surgical resection of primary LUAD in Shanghai Pulmonary Hospital from September 2015 to April 2016, including 27 males and 23 females, with a mean age of (66.24 ± 7.3) years. And all patients were followed up every three months for five years. Inclusion criteria: 1, all were diagnosed as lung adenocarcinoma by postoperative pathological examination; 2, all did not receive radiotherapy or chemotherapy before surgery; 3, clinical data were complete. Exclusion criteria: 1, combined with chronic systemic diseases; 2, combined with other malignant tumors. Written informed consents of all patients were obtained before the study. The study was approved by Shanghai Pulmonary Hospital Ethics Committee (ethical lot number: K21-111Y).

### Immunohistochemistry staining

After obtaining the tumor tissue, the tissue was routinely paraffin-embedded and preserved. In the experiment, tissue sections were dewaxed and rehydrated, and antigen repair was performed by incubating the slides in 10 mmol/L sodium citrate buffer and microwave treating the samples for 20 min. After being closed with 3% H2O2 and 10% normal goat serum (NGS), the slides were incubated with primary antibody at 4°C overnight. The paraffin-embedded LUAD sections were incubated with anti-TNFSF14 (ab115544, ABCAM), anti- JAM2 (ab156586, ABCAM), anti- LIFR (ab202847, ABCAM)、anti-SPN(ab101533, ABCAM)、anti- HGF(ab118871, ABCAM). The slides were then incubated with biotin-coupled anti-rabbit secondary antibody (1:1000, ab205718, Abcam, UK) for 2h at 37°C using the ABC kit from Vector Laboratories (Burlingame, CA, USA).

The sections were then incubated with polymeric HRP reagent and peroxidase activity was observed with diaminobenzidine tetrahydroxyl chloride solution (Vector Laboratories), and the sections were re-stained with hematoxylin. Ouant center is the analysis software of Pannoramic viewer. When the images of the tissue microarrays are scanned, the TMA software in Ouant center sets the numbers that correspond to the arrangement of the tissue sections. Thereafter, the densito quantification software in Quant center automatically identifies all dark brown dots in the microarray tissue as strongly positive, tan dots as moderately positive, light yellow dots as weakly positive, and blue cell nuclei only as negative, and analyzes the percentage of each stained (strong, moderate, weak, and negative) area in pixels, and finally performs an H-Score score.

### Bioinformatic and statistical analyses

All statistical analyses and graph plotting were performed using the R software (version: 4.04). Comparisons between the two groups were made using the Wilcoxon test and differences in proportions were compared using the chi-square test. The KM plotter was used to generate the survival curves and statistically significant differences were assessed using the log-rank test. Time-dependent ROC curves (tROC) were plotted using the R package ‘survivalROC’. Univariate and multivariate COX regression analyses were performed using the R package, “survival”. Additionally, ‘rms’ was used to plot nomograms and calibration curves, and decision curve analysis (DCA) was performed using the “DCA” package (33). Unless specified otherwise, two-tailed P < 0.05 denoted statistical significance.

## Results

### CAF clustering and identification of FRGs

**Figure 1:**
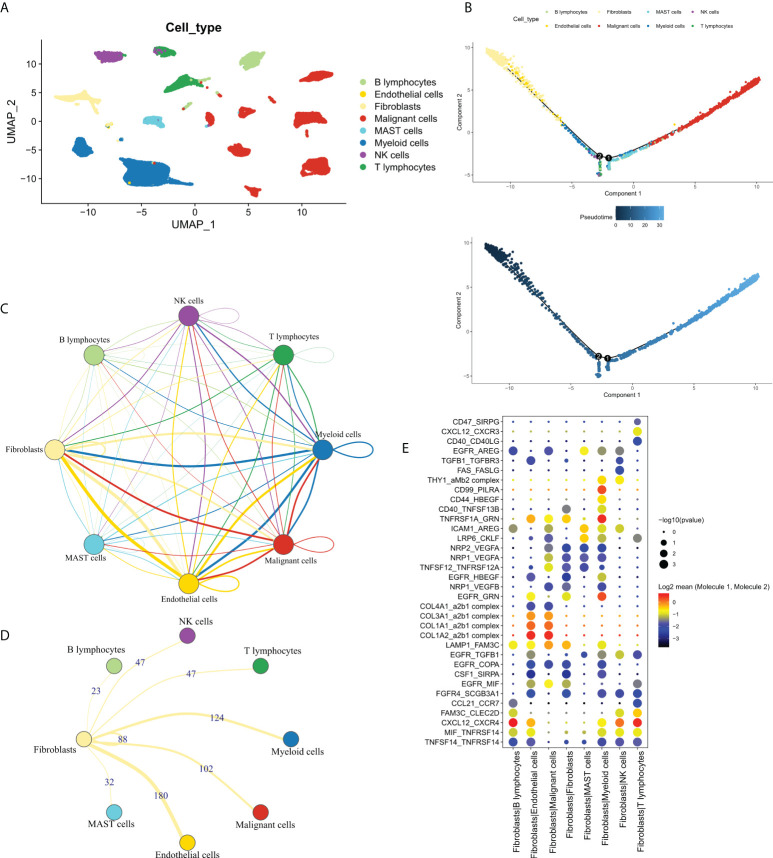
The single-cell profile of CAFs. **(A)** Eight cell clusters identified by cell clustering; **(B)** Pseudo-time analysis of the eight-cell clusters. Upper panel: cell cluster distribution, lower panel: pseudo-time distribution; **(C)** Communication network of the eight-cell clusters; **(D)** Communication network of CAFs with other cells, wherein the numbers represent the number of receptor-ligand pairs; **(E)** The top 5 receptor-ligand pairs of CAFs communicating with other cell types.

In order to explore the cross-talk between CAF and other cells and identifying the FRGs. We first analyzed the dataset, GSE131907, at single-cell resolution and identified a total of eight-cell clusters according to their original annotation (**Figure 1A**). The pseudo-time analysis suggested that CAFs were mainly aligned at the beginning of the trajectory (**Figure 1B**). Subsequently, the communication network between the eight-cell clusters was analyzed (**Figure 1C**). Specifically, CAFs were found to communicate the most with endothelial cells, followed by myeloid cells **(** **Figure 1D**). Significant receptor-ligand pairs were obtained as FRGs for subsequent analysis based on a set threshold of P < 0.05. The Dot plot was used to visualize the top five receptor-ligand pairs between CAFs and other cells (**Figure 1E**). Detailed results were shown in **Table S2**.

**Figure 2:**
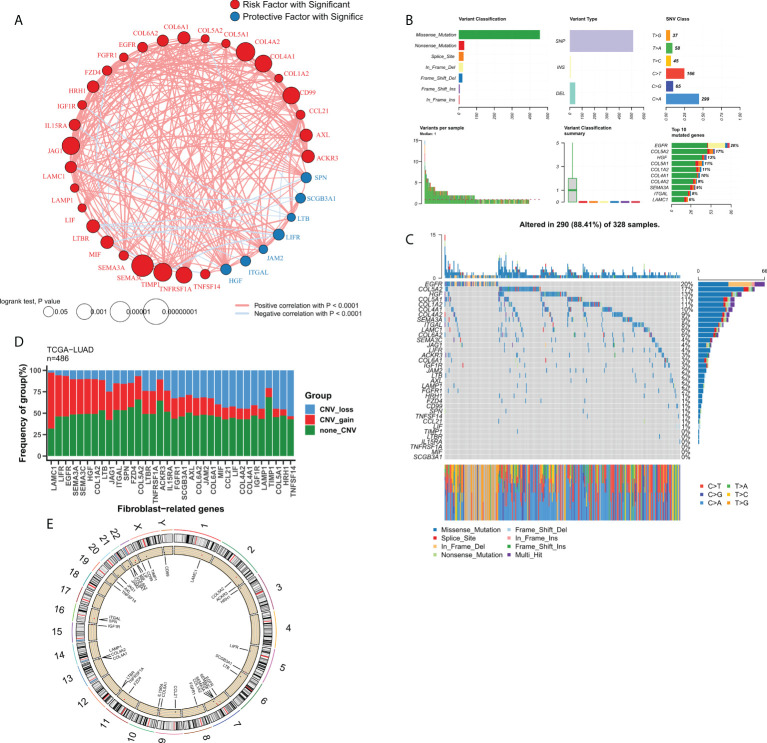
Genomic profile of FRGs in LUAD. **(A)** Correlation network of FRGs; **(B)** Summary of mutational events for FRGs in TCGA-LUAD; **(C)** Oncoplot showing the mutational mapping of FRGs; **(D)** Summary of CNV events for FRGs in TCGA-LUAD; **(E)** A loop graph showing CNV events for FRGs on chromosomes.

We then focused on FRGs with independent prognostic values. Univariate Cox analysis showed 33 independent prognostic factors in 127 FRGs. The loop graph in **Figure 2A** shows the correlation network and hazard ratios (HRs) for these 33 FRGs. **Figure 2B** displays the mutational landscape of the 37 FRGs. EGFR and COL5A2 were the top two genes with the highest mutation frequencies. The most common mutation was the missense mutation, whereas single nucleotide point mutation was the most common type of mutation, with the most frequently occurring change being from cytosine to adenine. The waterfall plot in **Figure 2C** shows the mutational landscape of 16 FRGs in patients. The bar chart displays the CNV profile of the 37 FRGs in TCGA-LUAD. Furthermore, FRGs underwent extensive CNV events. LAMC1 and TNFSF14 were the genes that experienced the most amplification and deletion events, respectively (**Figure 2D**). The loop graph was plotted to visualize the overall CNV profile of the 37 FRGs on the chromosomes (**Figure 2E**).

### Construction of an FRG-related risk model

**Figure 3:**
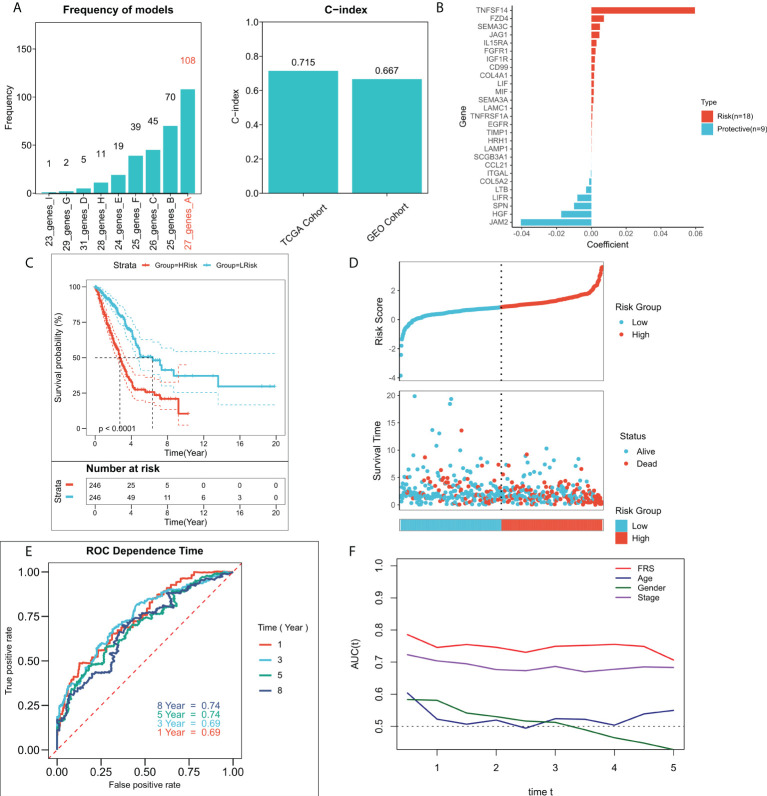
Construction of the FRG-related risk model. **(A)** Screening for the best LASSO model. Left panel: frequency of different gene combinations in the LASSO Cox regression model; Right panel: C-index of the best model in both TCGA and GEO cohorts; **(B)** LASSO coefficients for the 27 model genes; **(C)** KM survival curves for the high-FRS and low-FRS groups in the TCGA cohort; **(D)** Survival status and FRS of patients in TCGA cohort; **(E)** 1-, 3-, 5-, and 8-year ROC curves for FRS in TCGA cohort; **(F)** tROC curves for FRS and clinical characteristics in TCGA cohort.

An FRG-related risk model was constructed using the 37 FRGs with a prognostic value, on which 300 iterations of LASSO regression were performed. Of all the five combinations, the model containing seven genes was found to be the most stable and showed good accuracy in both the training and validation cohorts (TCGA: 0.715; GEO: 0.667) (**Figure 3A**). This LASSO model was constructed based on the optimal λ value of 0.01608, and FRS was calculated based on the following equation:


$$FRS=\sum i\;Coefficient\left(mRNA_{i}\right)\times Expression\left(mRNA_{i}\right)$$

shows the LASSO coefficient**Figure 3B** shows the LASSO coefficients for the model genes, detailed coefficients of 27 FRGs can be found in **Table S3**. Patients at high- and low-risk were distinguished based on the median FRS. Survival analysis suggested that patients in the high-risk group had significantly lower survival rates relative to those in the low-risk group (**Figure 3C**; P < 0.0001). **Figure 3D** shows the distribution of FRS in TCGA cohort and the transcriptional profiles of the model genes. 1, 3, 5, and 8-year AUC values for the model were 0.66, 0.67, 0.68, and 0.70 respectively (**Figure 3E**). The tROC analysis suggested that FRS and TNM staging were the best predictors (**Figure 3F**). Subsequently, the predictive efficacy of the model was also assessed in the validation set. The survival analysis suggested that patients in the high-FRS group showed significantly worse survival (**Figure S1A**; P < 0.0001). The ROC analysis suggested that the model had satisfactory predictive power in the external validation set, with 1, 3, 5, and 8-year AUC values of 0.68, 0.69, 0.69, and 0.71 respectively (**Figure S1B**). **Figure S1C** shows the distribution of FRS and model gene expression in the GEO cohort.

### Predictive independence of the risk model

**Figure 4:**
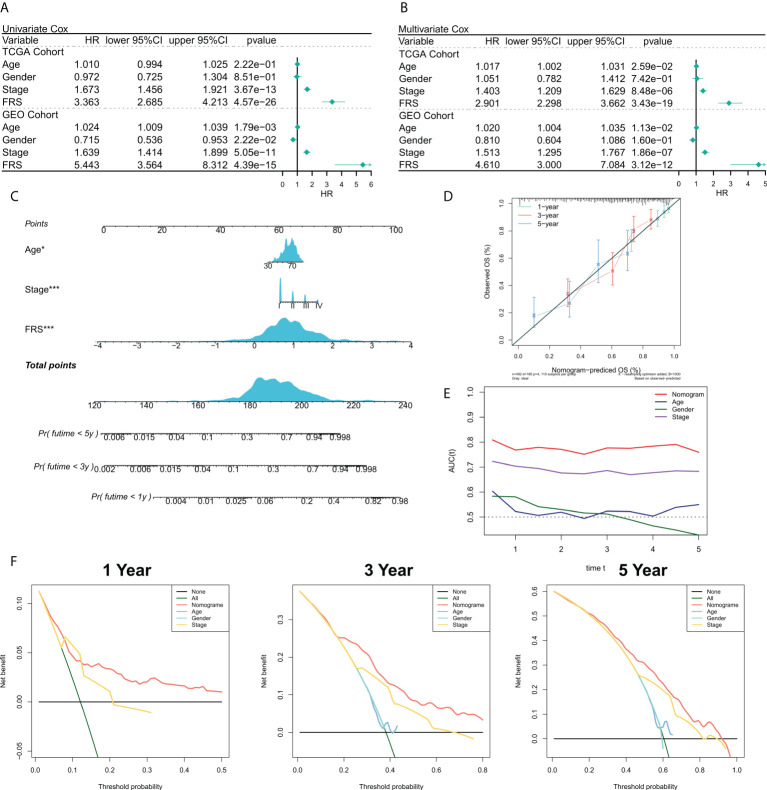
Validation of the FRG-related risk model. **(A)** Univariate Cox regression analysis of OS in TCGA and GEO cohorts; **(B)** Multivariate Cox regression analysis of OS in TCGA and GEO cohorts. **(C)** FRS-based nomogram; **(D)** Calibration curves for the nomogram; **(E)** tROC curves for the nomogram and clinical characteristics; **(F)** 1-, 3-, and 5-year DCA curves for the nomogram.

We then validate the prognosis value of the FRS model in the TCGA cohort and GEO meta cohort. The relationship between the risk scores and the clinical characteristics of the patients and their prognoses were analyzed using the univariate and multivariate Cox regression analyses. The results of the univariate Cox regression analysis suggested that FRS was an independent prognostic indicator in both the training and validation cohorts (P < 0.0001) (**Figure 4A**). The results of the multivariate Cox regression analysis showed that FRS remained an independent prognostic factor for overall survival (OS) in both the training and validation cohorts after correcting for other clinical characteristics (P < 0.0001) (**Figure 4B**). Furthermore, subgroup analysis indicated that FRS remained a reliable prognostic factor in different clinical groups (**Figure S2**). Therefore, risk scores could serve as a reliable prognostic marker for predicting OS in patients with LUAD. Subsequently, the nomogram was constructed to better assess the risk of patients with LUAD (**Figure 4C**). The correction curves for the nomogram showed a good 1-, 3-, and 5-year stability and accuracy of the nomogram model (**Figure 4D**). tROC analysis suggested that the nomogram model was a better predictor relative to the clinical characteristics (**Figure 4E**). Additionally, a DCA was conducted to assess the decision benefit of the nomogram model. The results showed that this nomogram model was suitable for 1-, 3-, and 5-year risk assessments of patients with LUAD (**Figure 4F**).

### Functional enrichment analysis of FRS

**Figure 5:**
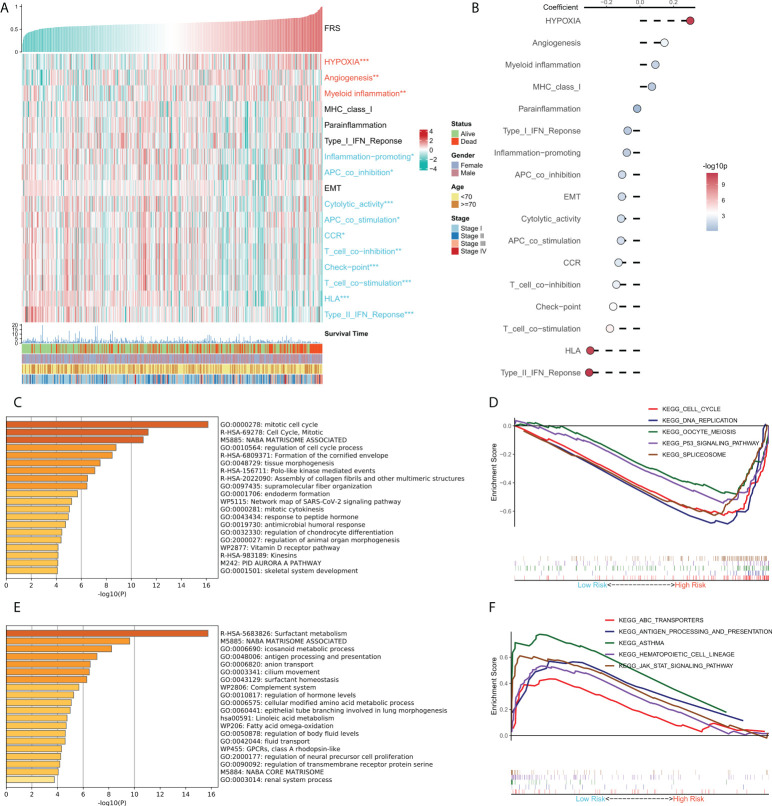
Functional analysis for FRS. **(A)** Heat map showing the correlation between FRS, biological pathway activities, and clinical characteristics; **(B)** Correlational analysis of FRS and biological pathways; **(C)** Functional enrichment analysis of the upregulated genes in the high group; **(D)** GSEA enrichment plot showing the five pathways of interest in the high group; **(E)** Functional enrichment analysis of the upregulated genes in the low group; **(F)** GSEA enrichment plot showing the five pathways of interest in the low group.

We tried to explain the potential biological logic of the differences in clinical outcomes among high- and low-FRS groups. Therefore, we assessed the correlation between FRS and some typical biological pathways. The heat map was plotted to illustrate the relationship among FRS, biological pathway activities, and clinical characteristics (**Figure 5A**), and the correlational analysis between FRS and biological pathways is shown on the right panel (**Figure 5B**). Angiogenesis, myeloid inflammation, and hypoxia were significantly positively correlated with FRS and their levels were markedly higher in the high-FRS group. GO analysis showed that the upregulated genes in the high-FRS group were mainly associated with the cell cycle, mitosis, and cytoskeleton (**Figure 5C**), whereas those in the low-FRS group were mainly related to antigen presentation and the complement system (**Figure 5E**). Further, GSEA showed that the cell cycle-related pathways such as the P53 signaling cascade, spliceosome, and DNA repair were significantly enriched in the high-risk group (**Figure 5D**), whereas antigen presentation, hematopoietic cell lineage, and the JAK-STAT signaling cascade were significantly enriched in the low-risk group (**Figure 5F**). Thus, these results suggested that tumor angiogenesis and DNA replication were active in the high-FRS group, whereas immune activity and immune cell differentiation were active in the low-FRS group.

### Immune landscape in the risk model

**Figure 6:**
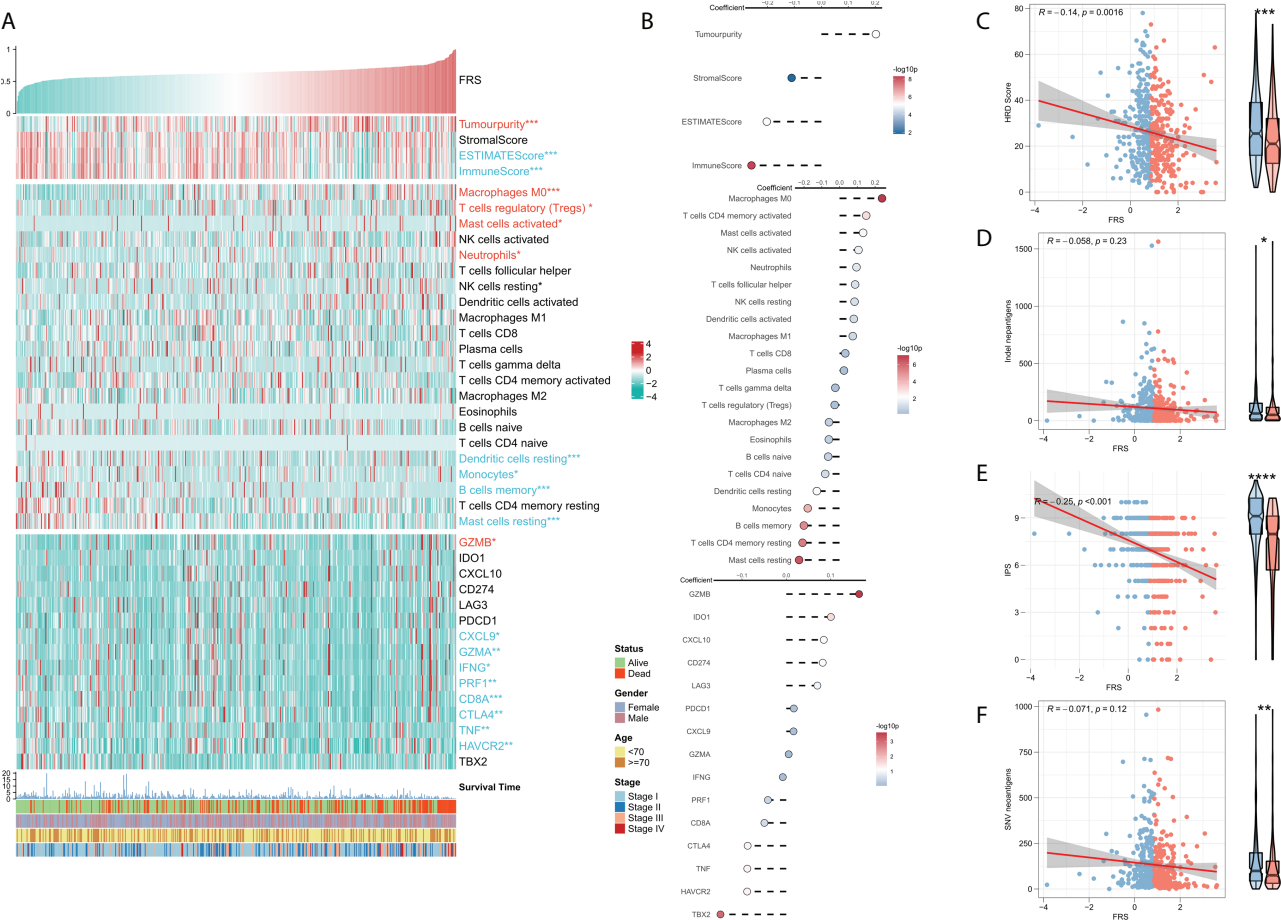
Immune infiltration analysis for FRS. **(A)** Heat map showing the correlation of FRS with the Estimate score, immune cell infiltration abundances, immune checkpoint expression, and clinical characteristics; **(B)** From top to bottom: correlational analysis of FRS with the Estimate score, immune cell infiltration abundances, and immune checkpoint expression. Scatter plot and box plot show the correlation of FRS with **(C)** HRD score; **(D)** Indel neoantigens; **(E)** IPS, and **(F)** SNV neoantigens.”Red name with * represents upregulated in high-risk score group, and blue name with * represents upregulated in low-risk score group; *p < 0.05; **p < 0.01; ***p < 0.001; ****p < 0.001.

TME plays a dual role in the tumorigenesis and progression of tumor and anti-tumor response. The correlation between FRS and the immune landscape was assessed in further detail. The heat map in **Figure 6A** demonstrates the relationship of FRS with the Estimate scores, abundances of immune-infiltrating cells, typical immune checkpoints (including CD274, CTLA4, HAVCR2, IDO1, LAG3, and PDCD1), immune active features (including CD8A, CXCL10, CXCL9, GZMA, GZMB, IFNG, PRF1, TBX2, and TNF), and clinical characteristics of the patients. The corresponding correlational analysis is shown on the right side of the heat map (**Figure 6B**). Tumor purity, M0 macrophages, and T regs were significantly positively correlated with FRS and these levels were significantly elevated in the high-FRS groups. In contrast, the Estimate score, immune score, DC cells, B cells, and monocytes were negatively correlated with FRS and these levels were significantly lowered in the low-FRS groups. Furthermore, the activities of CXCL9, GZMA, IFNG, PRF1, CD8A, CTLA4, TNF, and HAVCR2 were negatively correlated with FRS and enhanced in the low-FRS group. Subsequently, we focused on the four indicators related to tumor-specific antigens, including HRD score (**Figure 6C**), indel neoantigens (**Figure 6D**), IP S(**Figure 6E**), and SNV neoantigens (**Figure 6F**). The results showed that FRS was negatively correlated with the HRD score, IPS, and SNV neoantigens, and their levels were significantly elevated in the low-FRS group. These results suggested that patients in the low-FRS group experienced more chromosomal instability and had more tumor neoantigens, thereby contributing to a stronger immune system activity. Thus, we inferred that the patients with a low FRS may stand to gain more benefits from immunotherapy (34–36).

### Correlation between FRS and somatic variations

**Figure 7:**
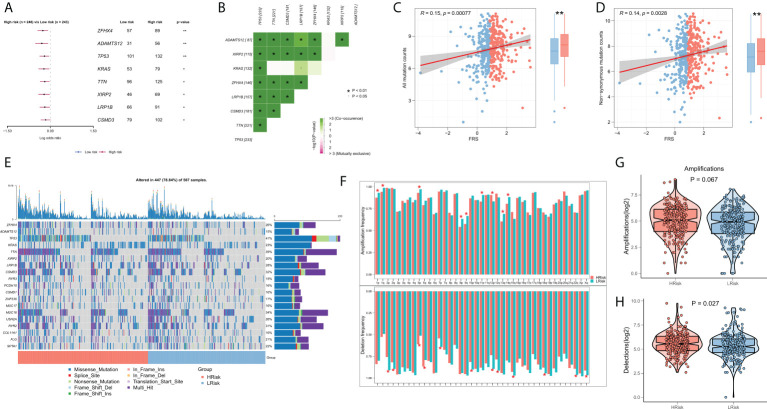
Genomic variation landscape of FRS. **(A)**Forest plots showing statistically significant differentially mutated genes between the high- and low-FRS groups; **(B)** Co-occurrence analysis of differentially mutated genes; **(C)** Correlation of FRS and all types of mutation burdens; **(D)** Correlation of FRS with non-synonymous mutation burden; **(E)** Oncoplot of high-frequency mutated genes between the high- and low-FRS groups; **(F)** Bar graph showing the CNV events on different chromosome arms in the high- and low-FRS groups; **(G)** Box plot showing the differences in the number of chromosome amplifications between high- and low-FRS groups; **(H)** Box plots showing the differences in the number of chromosomal deletions high- and low-FRS groups. *p < 0.05; **p < 0.01.

Several recent studies indicate that TMB is associated with patient responses to immunotherapy, whereby more somatic mutations may generate more potential mutation-derived antigens that can be recognized by the immune system. Further, the recognition of these antigens with mutant peptides by the immune system can activate immune functions and enhance anti-tumor immunity (37–39). Considering the clinical significance of TMB, we examined the correlation between TMB and FRS. The forest plot showed that the mutational frequencies of ZFHX4, ADAMTS12, TP53, KRAS, TTN, XIRP2, LRP1B, and CSMD3 were significantly greater in the high FRS-group (**Figure 7A**). The results of the mutation co-occurrence analysis suggested that the mutations in all the eight genes were highly co-occurring (**Figure 7B**). Correlation analysis showed that all mutation burdens and the non-synonymous mutation burden were significantly positively correlated with FRS and markedly increased in the high-FRS group (**Figures 7C, D**). **Figure 7E** details the mutational landscape of the high-frequency mutated genes in patients with LUAD. CNVs cause chromosomal variations differently. Thus, we further evaluated the correlation between FRS and CNV and found an increased frequency of amplifications and deletions in the low-FRS group at the level of the chromosome arm (**Figure 7F**). The box plots in **Figures 7G, H** show a significant increase in the chromosomal deletion events and an upward trend in amplification events in the high-FRS group.

### FRS-related guidance for clinical decision-making

**Figure 8:**
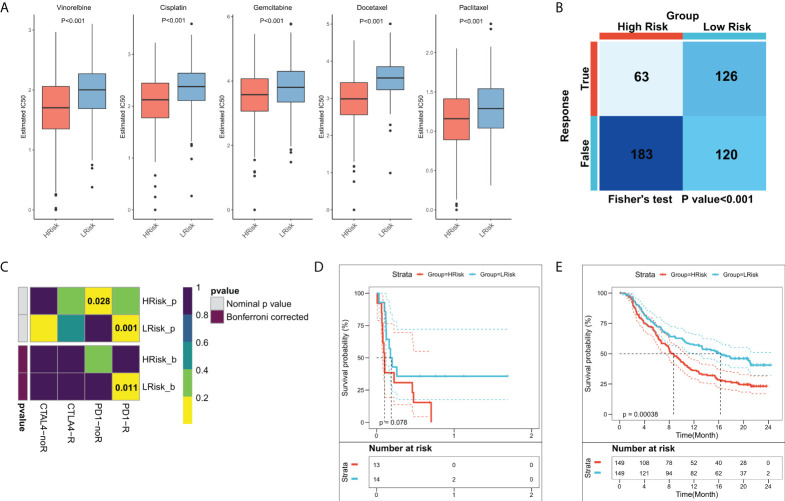
Role of the FRS-related risk model in guiding clinical treatment decisions. **(A)** Box plots showing the predicted IC50 values for the five most commonly used drugs in high- and low-FRS groups; **(B)** Predicted responses to immunotherapy for patients in the high- and low-FRS groups using the TIDE algorithm; **(C)** Sensitivity of patients in the high- and low-FRS groups to PD1 and CTLA4 treatment regimens predicted by subclass mapping; **(D)** KM survival curves for patients in the high- and low-FRS groups in GSE135222 cohort; **(E)** KM survival curves for patients in the high- and low-FRS groups in IMvigor210 cohort.

Previous results suggested that patients in different FRS groups have interesting differences in biological function, TME, and genomic variation, which may lead to different responses to chemotherapy and immunotherapy. Differences in patient sensitivities towards chemotherapeutic agents for LUAD were assessed and the results showed that patients in the high-FRS group in TCGA cohort were more sensitive to the commonly used five first-line agents (**Figure 8A**). The same results were observed in the validation cohort (**Figure S1D**). Overall, patients in the high-FRS group were more sensitive to chemotherapy. Based on the value of |log2 FC|, the top 300 differential expression genes between the high and low FRS groups were uploaded to the CMap database to search the underlying small molecular drugs. As shown in **Figure S3**, a total of 47 potential small molecular drugs were identified to target 35 biological process. Differences in the immune landscape and genomic alterations between the two groups suggested that FRS may be associated with immunotherapeutic efficacy. Therefore, we assessed the patient response rates to immunotherapy using the TIDE algorithm. The results showed a higher response rate to immunotherapy in the low-FRS group in TCGA cohort (**Figure 8B** **;** P = < 0.001). In the validation cohort, patients in the low-FRS group also responded substantially more to immunotherapy (**Figure S1E** **;** P < 0.001). The results of subclass mapping suggested that the patients in the low-FRS group were more sensitive to anti-PD1 therapy in both TCGA and GEO cohorts (TCGA: FDR = 0.011; GEO: FDR = 0.027) (**Figure 8C**; **Figure S1F**). Subsequently, we evaluated the prognostic performances of FRS in an immunotherapy cohort of NSCLC. The results showed that patients in the high-FRS group had a worse survival (**Figure 8D**; P = 0.078). Finally, we evaluated the utility of FRS in a large immunotherapy cohort, which also suggested that the patients in the high-FRS group had a significantly worse survival (**Figure 8E**; P = 0.00038),. Overall, these results demonstrated that the risk model constructed in this study was a powerful tool to guide decisions related to chemotherapy and immunotherapy for the treatment of LUAD.

### Validation of key FRGs in the clinical samples

**Figure 9:**
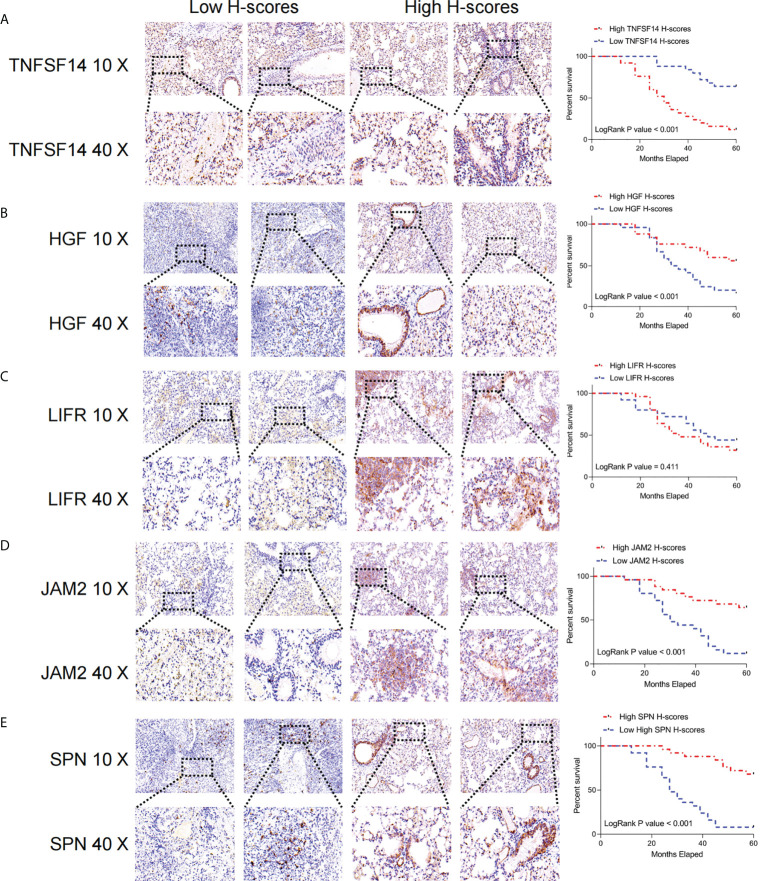
Immunohistochemical analysis of the key FRGs in the FRS model. High TNFSF14 expression, low JAM2, HGF, and SPN expression were associated with poor prognosis in patients with LUAD. **(A)** Immunohistochemical analysis of the intensity of TNFSF14 staining in tumors from 50 patients with lung adenocarcinoma and Kaplan-Meier analysis of the correlation between H-scores of immunohistochemistry for TNFSF14 and the survival cycle of patients with LUAD; **(B)** Immunohistochemical analysis of the intensity of HGF staining in tumors from 50 patients with lung adenocarcinoma and Kaplan-Meier analysis of the correlation between H-scores of immunohistochemistry for HGF and the survival cycle of patients with LUAD. **(C)** Immunohistochemical analysis of LFR staining intensity in tumors from 50 lung adenocarcinoma patients, Kaplan-Meier analysis of H-scores of immunohistochemistry for LFR correlated with the survival cycle of LUAD patients; **(D)** Immunohistochemical analysis of JAM2 staining intensity in tumors from 50 lung adenocarcinoma patients, Kaplan-Meier analysis of JAM2 H-scores of immunohistochemistry correlated with the survival cycle of LUAD patients; **(E)** immunohistochemistry analyzed the intensity of SPN staining in the tumors of 50 lung adenocarcinoma patients, and Kaplan-Meier analyzed the correlation of H-scores of immunohistochemistry of SPN with the survival cycle of LUAD patients.

We extracted the most representative top 5 genes according to lasso coefficient for external validation. The staining intensity of TNFSF14, JAM2, HGF, SPN, and LIFR in the tumors of 50 lung adenocarcinoma patients was first analyzed by immunohistochemistry and quantified according to H-scores. Subsequently, they were defined as the high expression group (H scores > median value) and low expression group (H scores < median value) according to the median value of H-scores, respectively. Subsequently, we performed a prognostic analysis of their Kaplan-Meier according to staining intensity, and we found that patients with higher staining intensity in TNFSF14 had a significantly worse prognosis and had shorter survival cycles **(** **Figure 9A** **)**. However, patients with higher staining intensity for JAM2, HGF, and LIFR had significantly higher survival cycles than those with lower staining intensity for lung adenocarcinoma **(** **Figures 9B, D, E** **)**, but for SPN, there was no significant correlation between their staining intensity and patients’ survival cycles **(** **Figure 9C** **).**

## Discussion

Considering the complexity of TME in LUAD patients, previous research attention was focused more on the immune cells, however, the crosstalk between CAFs and other cells remains far less understood. In this study, we used single-cell RNA sequencing data to assess the communication between CAFs and other cells and identified the interacting molecules. Subsequently, FRS was constructed from bulk sequencing data based on interacting genes and its significance in prognostic and therapeutic decision-making was determined. Functional enrichment analysis was employed to understand FRS-related biological functions. Additionally, CIBERSORT, ssGSEA, and ESTIMATE algorithms were used to map the TIME landscape and assess the associations between FRS and TIME by analyzing the LUAD-related genomic information. Finally, the intrinsic associations between FRS and genomic alterations were assessed in terms of tumor mutation burden and CNV effects.

CAF is a pro-tumor stromal cell component in most solid tumors. The interaction between CAFs and various cellular components in TME regulates tumor progression and invasion (13, 14, 40). In this study, we first examined the intercommunication between CAFs and other cells. The pseudotime analysis showed that the distribution of CAFs was mainly at the beginning of cell sorting trajectories, thus suggesting that CAFs were involved in the formation of stromal components in the early stages of cancer progression. Cellular communication analysis revealed additional interactions of CAFs with endothelial, malignant, and myeloid cells. These findings demonstrated that CAFs were not only involved in stromal formation and regulation of tumor progression but also interacted extensively with immune cells. Receptor-ligand analysis suggested that the CAFs could regulate immune cells mainly through the TNF signaling pathway. Subsequently, the significant receptor-ligand pairs were identified as FRGs and an FRG-based FRS model was generated using the LASSO algorithm. This model showed excellent predictive performances in both the training and the external validation cohorts and suggested a significant deterioration in survival among the high-risk patients.

Patients with LUAD have a high tumor mutation burden and show strong immunogenicity. Therefore, LUAD is an ideal indication for immunotherapy (41). However, the overall response rate of patients towards immunotherapy is low and only a certain proportion of patients benefit from it (42). Therefore, identifying “hot” tumors in LUAD is expected to enhance the decision and selection of those who would benefit from immunotherapy. In the present study, functional enrichment analysis suggested that a high FRS was associated with hypoxia, angiogenesis, and myeloid immunity; among them, hypoxia is often considered a limiting factor for TME and can lead to treatment resistance (43). Angiogenesis is essential for tumor growth and metastases, and both angiogenesis and myeloid immunity are inhibitors of the immune system functions (44–47). The activities of most anti-tumor immune and antigen-presentation pathways were markedly increased in patients with a low FRS. The interaction between TIME and immune cells is closely related to immunotherapy and patient prognoses (48–50). We further analyzed the abundances of immune infiltrates in TME and the findings suggested that a high FRS was associated with a higher tumor purity and an elevated Treg level, ultimately leading to immunosuppression (51). In contrast, patients in the low FRS group had higher immune scores, an increased proportion of DC cells, and enhanced immune checkpoint activities. Further, the immunophenotype scores were found to be negatively correlated with FRS and were markedly high in the low-FRS group. These results suggested a low FRS-activated immunophenotype (29, 52), consistent with the better survival of the patients in the low-FRS group and the resultant development of “hot” tumors sensitive towards immunotherapy. Additionally, HRD scores, indel neoantigens, and SNV neoantigens were elevated in the low-FRS group. These findings suggested that more tumor-specific neoantigens may be present in patients in the low-FRS group and they may be more likely to benefit from immunotherapy (53–55).

A recent study shows that genomic alterations are closely related to neoantigen formation and immunotherapeutic responses (56). However, the results of this study suggest that patients in the low-FRS group experience less TMB and that the high-frequency mutated genes were mutated more frequently in the high-FRS group. To elucidate this phenomenon, the mutational co-occurrence of the high-frequency mutated genes was examined. As these were all highly co-mutated genes, patients in the high-FRS group showed a higher TMB frequency. Furthermore, patients in the low-FRS group experienced a higher frequency of CNVs in the chromosomal arms but fewer CNV events in total. These results suggested that FRS could better reflect the immune status of the tumor and predict the patient responses to immunotherapy relative to TMB and CNV.

In summary, low FRS resulted in “hot” tumors with an immune-activating phenotype and possibly the production of more tumor neoantigenic peptides. We then systematically evaluated the patient responses to chemotherapy and immunotherapy. Patients with a high FRS were more sensitive to chemotherapy. Previous functional enrichment results suggested that the cell cycle-related pathways, as targets for chemotherapy, were active in patients with a high FRS, thereby leading to better chemotherapeutic benefits. Subsequently, the TIDE and subclass mapping algorithms predicted a higher patient sensitivity towards anti-PD1 therapy in those with a low FRS, consistent with our previous findings. Moreover, we observed better survival in patients with a low FRS in both the external NSCLC immunotherapy and the large immunotherapy cohorts. Overall, these results demonstrated that the FRS model is a powerful tool that can guide the treatment-decision making for patients with LUAD. Patients with a high FRS are better suited for chemotherapy, whereas those with a low FRS are more likely to benefit from immunotherapy.

However, the present study has some limitations. First, the similarity of expression profiles of CAFs and vascular cells may confound our analysis due to the lack of finer cell classification. Second, bulk sequencing only reflects inter-patient heterogeneity and not intra-tumoral heterogeneity. Third, although we have employed several algorithms to assess the accuracy of this FRS model for predicting patient sensitivity towards chemotherapy and immunotherapy, further validation of these findings by prospective cohort studies and clinical data is required. Finally, additional *in vivo* and *in vitro* experiments should be performed to confirm the specific mechanisms underlying the crosstalk of FRGs with other cells in CAFs, which are expected to contribute to the further understanding of the functions of these CAFs.

In summary, this study contributed towards the understanding of cellular interactions in CAFs and TME, and we developed a novel, FRS-based model. This model allowed for the systematic quantification of “cold” and “hot” tumor patterns from multiple perspectives, including function, immune infiltration, and genomic alterations. Moreover, it can also facilitate the quantitative estimation of patient prognoses and guide the clinical decision-making for chemotherapy and immunotherapy.

## Data availability statement

The original contributions presented in the study are included in the article/**Supplementary Material**. Further inquiries can be directed to the corresponding author.

## Ethics statement

The study was approved by Shanghai Pulmonary Hospital Ethics Committee (K21-111Y). The patients consented to participate.

## Author contributions

SW analyzed the data and wrote the manuscript. XG carried out data interpretations and helped data discussion. WZ conceived and designed the whole project and drafted the manuscript. All authors read and approved the final manuscript.

## Funding

This work was supported by the Youth Found of National Natural Science Foundation of China (82103309).

## Acknowledgments

The authors hereby express their gratitude to all participants who supported the study.

## Conflict of interest

The authors declare that the research was conducted in the absence of any commercial or financial relationships that could be construed as a potential conflict of interest.

## Publisher’s note

All claims expressed in this article are solely those of the authors and do not necessarily represent those of their affiliated organizations, or those of the publisher, the editors and the reviewers. Any product that may be evaluated in this article, or claim that may be made by its manufacturer, is not guaranteed or endorsed by the publisher.

## Supplementary material

Supplementary Figure 1 External validation of FRS **(A)** KM survival curves for patients in the high- and low-FRS groups in the GEO cohort; **(B)** 1-, 3-, 5-, and 8-year ROC curves for FRS in the GEO cohort; **(C)** Survival status and FRS of patients in the GEO cohort; **(D)** Box plots showing the predicted IC50 values of the five most commonly used drugs in high- and low- FRS groups in the GEO cohort; **(E)** Immunotherapeutic responses of patients in the high- and low-FRS groups in the GEO cohort predicted using the TIDE algorithm; **(F)** Sensitivity of the patients in the high- and low-FRS groups to PD1 and CTLA4 treatment regimens in the GEO cohort predicted using the subclass mapping algorithm.

Supplementary Figure 2 Subgroup Cox analysis of FRS Subgroup Cox regression analysis of FRS in TCGA **(A)** and GEO **(B)** cohorts.

Supplementary Figure 3 Prediction of FRS-related small molecule compounds.
