# Supplementary figures and images for: Development and validation of a novel fibroblast scoring model for lung adenocarcinoma
Source: Front Oncol. 2022 Aug 22;12:905212. doi: 10.3389/fonc.2022.905212 (PMC9444064; doi:10.3389/fonc.2022.905212)

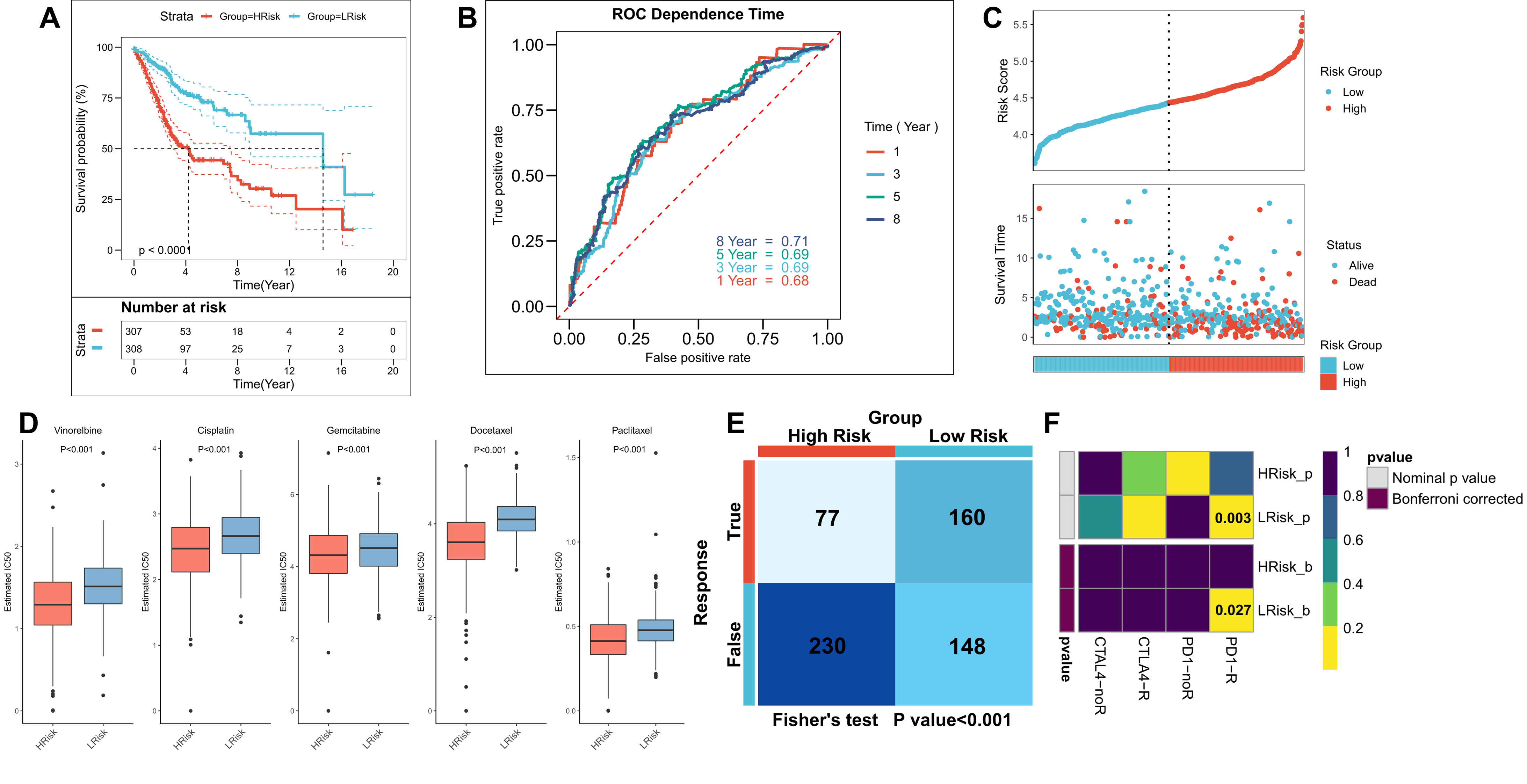

Supplement: Supplementary Figure 1 — External validation of FRS (A) KM survival curves for patients in the high- and low-FRS groups in the GEO cohort; (B) 1-, 3-, 5-, and 8-year ROC curves for FRS in the GEO cohort; (C) Survival status and FRS of patients in the GEO cohort; (D) Box plots showing the predicted IC50 values of the five most commonly used drugs in high- and low- FRS groups in the GEO cohort; (E) Immunotherapeutic responses of patients in the high- and low-FRS groups in the GEO cohort predicted using the TIDE algorithm; (F) Sensitivity of the patients in the high- and low-FRS groups to PD1 and CTLA4 treatment regimens in the GEO cohort predicted using the subclass mapping algorithm. [file Image_1.tif]

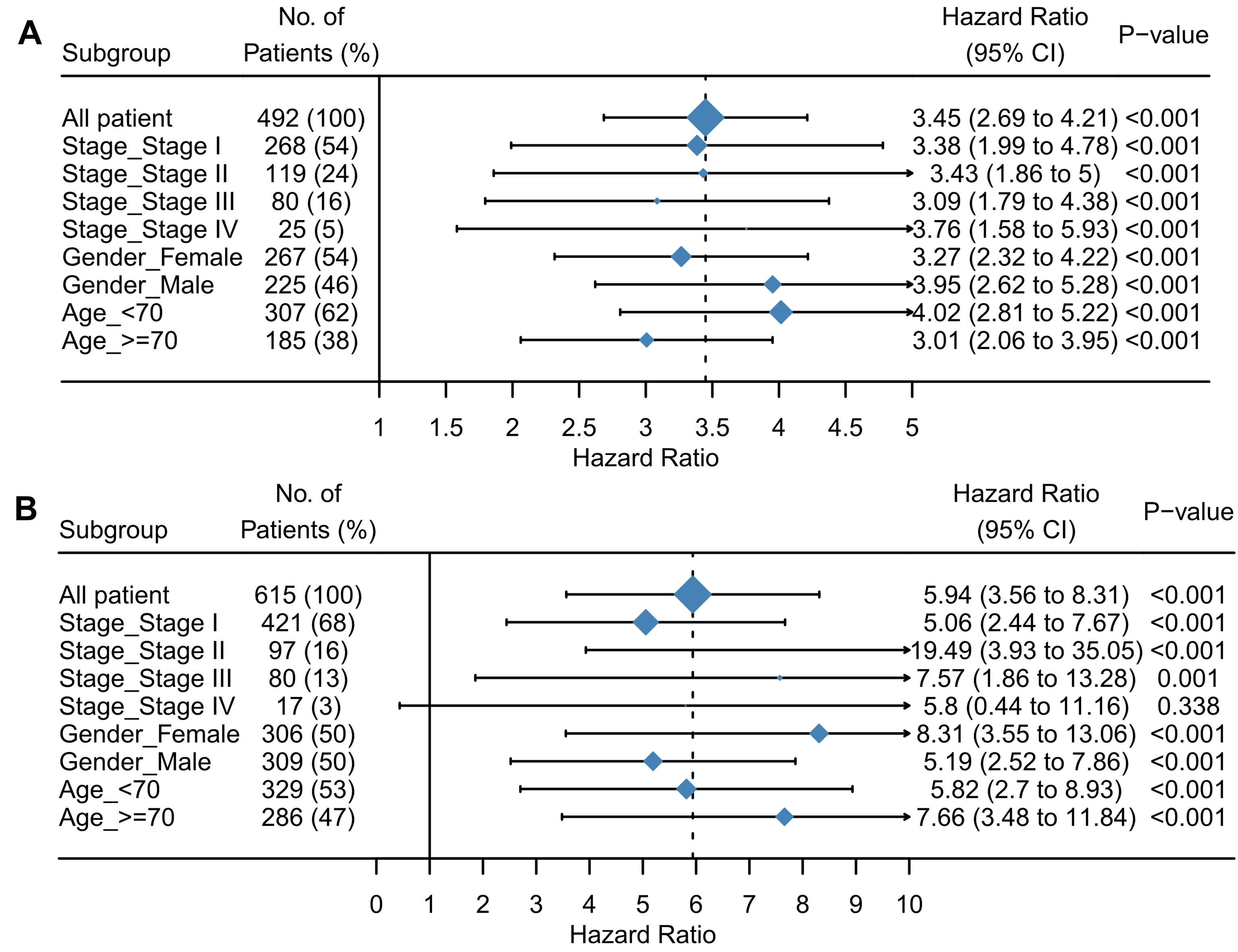

Supplement: Supplementary Figure 2 — Subgroup Cox analysis of FRS Subgroup Cox regression analysis of FRS in TCGA (A) and GEO (B) cohorts. [file Image_2.tif]

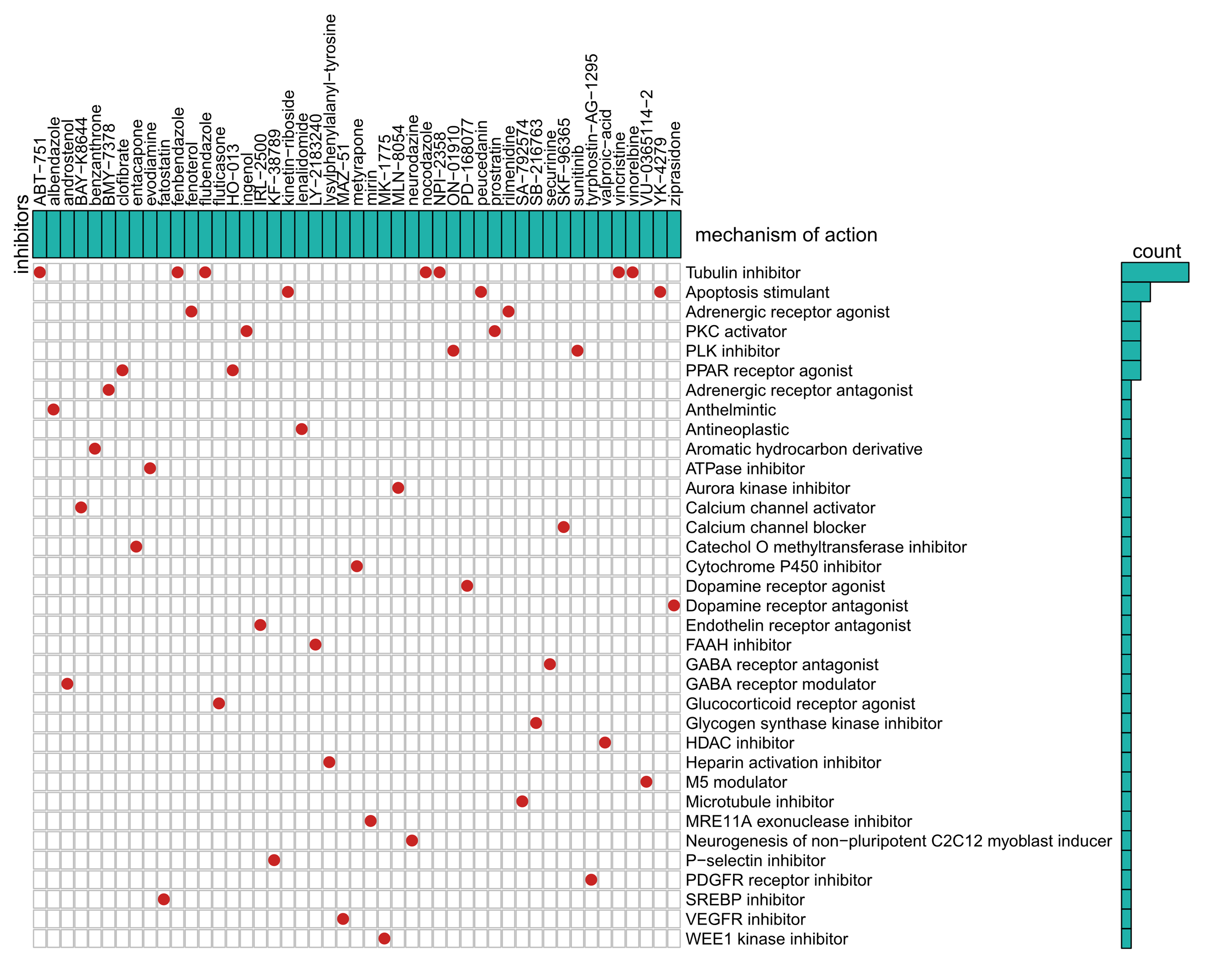

Supplement: Supplementary Figure 3 — Prediction of FRS-related small molecule compounds. [file Image_3.tif]
